# Survival Outcomes in Early Onset Appendiceal Adenocarcinoma (EOAA)

**DOI:** 10.1245/s10434-026-19855-z

**Published:** 2026-06-06

**Authors:** Rushabh Gujarathi, Erika Belmont, Christopher Rodman, Varun Vivek Bansal, Namrata Setia, Lindsay Alpert, John Hart, Mecker G. Möller, Oliver S. Eng, Grace Lee, Janet Chin, Manik A. Amin, Blase N. Polite, Chih-Yi Liao, Kiran K. Turaga, Ardaman Shergill

**Affiliations:** 1 Department of Medicine, University of Louisville, Louisville, USA; 2 Department of Medicine, Washington University School of Medicine, St. Louis, USA; 3 Department of Medicine, Yale School of Medicine, New Haven, USA; 4 Department of Surgery, University of Colorado, Aurora, USA; 5 Department of Pathology, University of Chicago, Chicago, USA; 6 Department of Surgery, University of Chicago, Chicago, USA; 7 Department of Surgery, University of California, Irvine, Orange, USA; 8 Department of Radiology, University of Chicago, Chicago, IL USA; 9 Department of Medicine, Section of Hematology/Oncology, University of Chicago, University of Chicago Medicine, Chicago, IL USA; 10 Department of Surgery, Yale School of Medicine, New Haven, CT USA

## Abstract

**Background:**

The incidence of early onset (age < 50 years at diagnosis) appendiceal adenocarcinoma (EOAA) is rising alarmingly. Reported data on etiology, treatment, and outcomes is scarce. In this study, we report clinical outcomes for patients with EOAA.

**Patients and Methods:**

Patients diagnosed with appendiceal adenocarcinoma between 2013 and 24 were stratified on the basis of age at diagnosis as having either EOAA (< 50 years) or average-age onset appendiceal adenocarcinoma (AOAA; ≥ 50 years). Clinicopathological and genomic data were abstracted from electronic medical records. Baseline clinicopathologic features and survival outcomes were compared between patients with EOAA and AOAA.

**Results:**

Of 181 eligible patients, 49 (27.1%) had EOAA. Median age at diagnosis for patients with EOAA was 42.1 years (interquartile range [IQR], 35.7–46). Of the 76 patients (EOAA = 23 [30.3%] + AOAA = 53 (69.7%)] who had locoregional disease at presentation, 44 (57.9%) remained disease-free for at least 2 years. Within these 44 (conditional survival), the EOAA group showed numerically shorter recurrence-free survival (RFS) and higher risk of recurrence (median RFS, EOAA = 49.5 months versus AOAA = 83.3 months; hazard ratio [HR], 4.07; 95% confidence interval [CI], 1.49–11.18;* p *= 0.06). Long-term recurrences (≥ 5 years) were seen in 9/15 (60%) patients during the 5-year follow-up period. Of 139 evaluable patients (EOAA = 36 [25.9%] + AOAA = 103 [74.1%]) with metastatic/recurrent disease, patients with EOAA received more non-fluorouracil (FU)-based systemic treatments, including experimental agents, in any line of treatment (8/36, 22.2% versus 6/103, 5.8%;* p *= 0.009). A higher proportion of patients in the EOAA group received bevacizumab in any line of treatment (24/36, 66.7% versus 49/103, 47.6%;* p *= 0.055) and three or more lines of systemic therapy (10/36, 27.8% versus 14/103, 13.6%;* p *= 0.07). There was no significant difference in OS between the EOAA and AOAA groups despite more aggressive therapy in EOAA (median OS, EOAA = 35.2 months versus AOAA = 40.6 months; HR, 0.90; 95% CI, 0.57–1.44;* p *= 0.66).

**Conclusions:**

Among patients who remained disease-free after initial surgical resection for locoregional disease for at least 2 years, recurrence was more frequent in patients with EOAA. Aggressive systemic therapy, including trials and non-FU based therapies were more frequent in patients with EOAA but were not associated with improved survival. EOAA may represent a unique entity within this rare disease, which warrants further exploration.

**Supplementary Information:**

The online version contains supplementary material available at 10.1245/s10434-026-19855-z.

Appendiceal cancers (ACs) represent a rare malignancy with an incidence of less than 2 cases per 100,000 person-years; however, their incidence has been increasing in the USA in recent years.^1,2^ In a recent Surveillance, Epidemiology, and End Results (SEER) study analyzing early onset (age < 50 years at diagnosis) malignancies, the fastest growing incidence among gastrointestinal cancers was noted for cancers originating in the appendix, with a 15.6% mean annual percentage change between the years 2010 and 2019.^3^ The age-standardized incidence rate of ACs per 100,000 early onset cancers increased from 0.33 in 2010 to 1.13 in 2019.^3^ While much of this rise is thought to be driven by neuroendocrine tumors, appendiceal adenocarcinomas have also demonstrated increasing incidence.^1,2^

Nearly 20% of early onset solid tumors, conventionally considered to be cancers diagnosed below 50 years of age, demonstrate frequent associations with germline mutations.^4–7^ However, this landscape remains incompletely characterized in the case of ACs, although recurring germline mutations in genes such as *MUTYH* and *MLH1* have been reported in some studies.^8^ There have been suggestions of a lack of a contributory role of germline mutations as drivers of malignancy even when these are found in patients with appendiceal adenocarcinoma (AA).^9^ Analyses of somatic mutations have revealed that *PIK3CA*, *SMAD3*, *TSC2* (all more frequent in patients aged < 50 years at diagnosis), and *GNAS* (more frequent in patients aged ≥ 50 years at diagnosis) alterations may have differential incidence in patients with AA based on age at diagnosis.^10^ Hence, along with the known burdens of an early onset cancer diagnosis such as significant physical, psychosocial, and financial stress, patients with early onset ACs may also face unique challenges owing to the overall rarity and incomplete understanding of the disease.^11,12^ Therefore, there is a need to accurately characterize the clinical, genomic, and biologic features of early onset ACs.

Despite recent studies highlighting distinctive characteristics in patients with early onset ACs, there remains a dearth of evidence regarding care patterns and treatment outcomes in this patient population.^13^ To address this gap, we examined the clinicopathological characteristics, treatment outcomes, and genomic features in patients with early onset appendiceal adenocarcinoma (EOAA) seen at a single high-volume cancer center. We present a comparison of disease features and outcomes in patients with EOAA and average-age onset appendiceal adenocarcinoma (AOAA) seen at our institution between 2013 and 2024.

## Patients and Methods

### Patient Selection

We conducted a review of medical records to identify patients with EOAA seen at the University of Chicago between November 2013 and May 2024. Date of data cutoff was 28 May 2024. The review of records for this study was approved by the Institutional Review Board at the University of Chicago, and a waiver of consent was granted (IRB24-0934). Patients with mucinous adenocarcinomas (MAs), goblet cell adenocarcinomas (GCAs), colonic-type (nonmucinous) adenocarcinomas, signet ring cell adenocarcinomas (SRCAs), and poorly differentiated adenocarcinomas were included. Patients with GCAs were included as the natural history of patients with GCAs resembles high-grade Aas, and similar management is often recommended.^14–18^ Cases with well-differentiated neuroendocrine tumors, and low- or high-grade appendiceal mucinous neoplasms (LAMN/HAMN) were excluded since these diseases are often managed differently compared with AAs.^19^

Consecutive adult (age 18 years and above at diagnosis) patients with histologic confirmation of AA were included. Patients were considered to have EOAA if the age at diagnosis was below 50 years (18–49 years) and AOAA if the age at diagnosis was more than or equal to 50 years. A minimum of 1-year follow-up after first treatment was required to allow adequate follow-up time. For the purposes of our report, one “line” of treatment was defined as the period from initiation of any systemic intervention(s) until the time of clinically determined disease progression on that regimen, which warranted a different intervention, or death, whichever was earlier.^20^ Hence, if patients received a therapeutic regimen as first-line therapy and never had progression of disease, but were continued on modified maintenance therapy (which may sometimes be seen in the management of gastrointestinal cancers), the length of initial treatment regimen plus maintenance regimen was considered as a single line of treatment.

Patients without evidence of distant metastases at the time of diagnosis were considered to have locoregional disease (LRD). Patients who presented with distant metastases at diagnosis (synchronous) or disease recurrence after initially presenting with LRD (metachronous) were included in the metastatic/recurrent disease (M/R) cohort. This is in line with current treatment guidelines where similar treatment is recommended for peritoneal metastatic recurrence and de novo metastatic disease.^21^

#### Outcomes

The primary outcomes analyzed were recurrence-free survival (RFS) in patients with LRD, and overall survival (OS) in patients with M/R disease. RFS was calculated from the date of curative-intent hemicolectomy to date of confirmed disease recurrence or death, whichever was earlier. For patients who did not undergo curative-intent right hemicolectomy, RFS was calculated after appendectomy or last definitive oncologic surgery. OS was calculated from the date of diagnosis of M/R disease to date of death, with patients being censored at their date of last clinical contact if alive at the date of data cutoff. For patients with LRD who had recurrence at a later time, the date of confirmed histopathologic recurrence was the date of M/R disease diagnosis. Specifically, the date on which disease recurrence was first identified on imaging was considered as the date of recurrence in this group. Patients who did not have disease recurrence by data cutoff were censored for RFS at the date of last available stable imaging. Conditional survival analyses were conducted at the 1-, 2-, and 3-year marks after the abovementioned surgeries owing to the crossing of Kaplan–Meier curves between the 2- and 3-year marks in the initial RFS analysis, with a visual depiction of conditional analysis presented for the 2-year mark.

#### Tumor Genomics and Germline Data

Tumor genomic data from next-generation sequencing (NGS) were included when available from a Clinical Laboratory Improvement Amendments (CLIA)-certified laboratory, including the University of Chicago’s in-house Oncoplus NGS panel.^22^ Variants of uncertain significance were not included in this report.

#### Statistical Methods

Unless otherwise specified, continuous variables are reported as medians and interquartile ranges (IQR). Categorical variables are reported as percentages. Continuous variables were compared between disease groups using the Wilcoxon rank-sum test for independent samples. Proportions for categorical variables (including treatment patterns) were compared using Fischer’s exact test. Variables with *p*-values < 0.1 were considered to show meaningful signals and further explored using multivariable logistic regression analysis to adjust for relevant clinicopathological characteristics. Associations between study outcomes, disease subgroups, and clinicopathological characteristics were studied using Cox proportional hazards regression analysis. Kaplan–Meier estimations were used to report median (m) survival times. The threshold for statistical significance for all tests was set at *p *< 0.05. Statistical analyses were performed using BlueSky Statistics (version 10.3.2) and STATA (version 18.0).

## Results

### Selection of Records

**Fig. 1:**
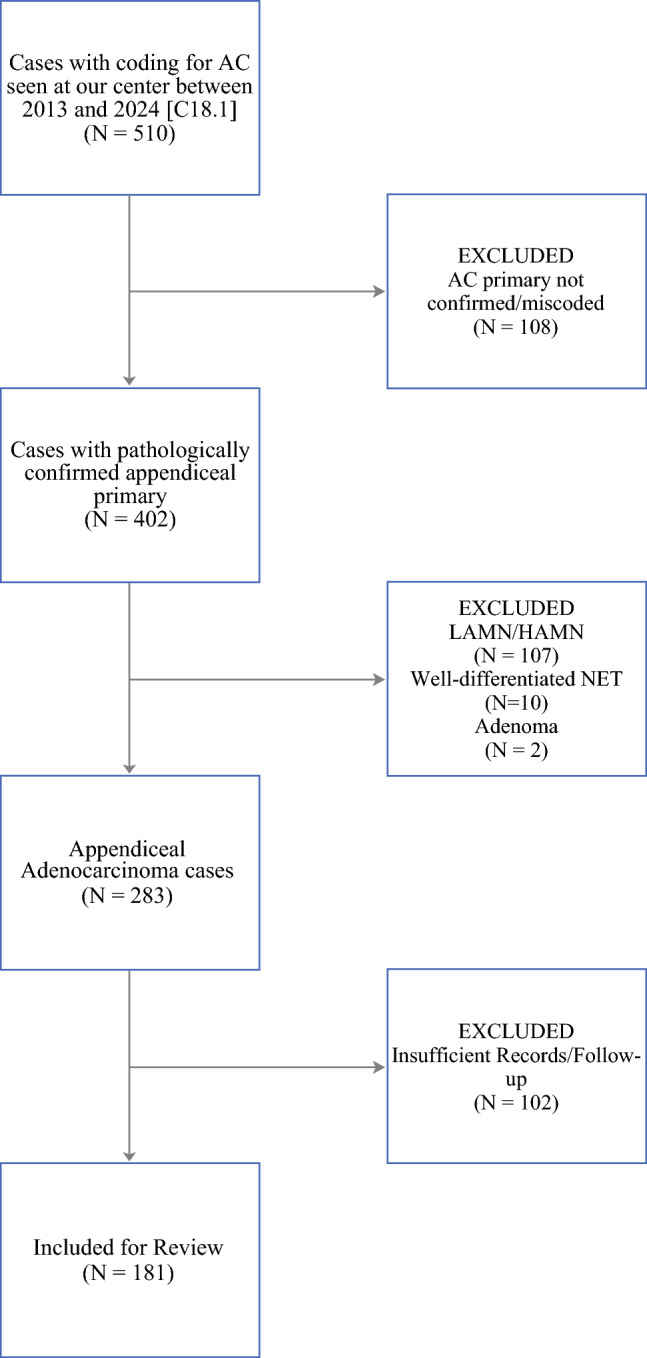
STROBE flowchart for subject selection AC appendiceal cancer, C18.1 International Classification of Diseases, Tenth Revision, coding for appendiceal cancer, LAMN/HAMN low-grade/high-grade appendiceal mucinous neoplasm, NET neuroendocrine tumor, STROBE Strengthening the Reporting of Observational Studies in Epidemiology

A total of 181 patients were included in our analysis, of which 49 (27.1%) had EOAA. The selection process for individual analyses per the inclusion criteria (see Methods) for the study is shown in (Fig. 1). The process for the subanalyses is shown in (Supplementary Fig. 1).

### Clinicopathologic and Demographic Characteristics

Patient demographics and clinicopathological characteristics at diagnosis, stratified on the basis of age group, along with group comparisons, are presented in (Table 1). The proportions of female patients presenting with ovarian metastases (among female patients with ovaries present at the time of AC diagnosis) was noted to be 59.3% in the EOAA group and 38.1% in the AOAA group (16/27 versus 24/63;* p *= 0.06). Adjusting for histology (GCA versus non-GCA), grade, and lymph node disease in a multivariable logistic regression model (Supplementary Table 1), female patients (with ovaries present) in the EOAA group showed higher odds of having ovarian metastases at presentation, albeit with no statistically significant association (odds ratio [OR], 2.36; 95% confidence interval [CI], 0.93–6.20;* p *= 0.07).

**Table 1:** Demographics and clinicopathological characteristics of the study population, stratified by age at onset

Characteristic (percentage of group total)	Entire cohort	EOAA	AOAA	*p*-Value
Total number of patients	181	49	132	
Median age at diagnosis in years (IQR)	57.6 (48.2–66.3)	42.1 (35.7–46)	61.7 (56.7–68.5)	
Sex assigned at birth				0.87
Female	102 (56.4%)	27 (55.1%)	75 (56.8%)	
Male	79 (43.6%)	22 (44.9%)	57 (43.2%)	
Race				0.41
White	139 (76.8%)	36 (73.5%)	103 (78.0%)	
Black or African American	31 (17.1%)	8 (16.3%)	23 (17.4%)	
Asian/Mideast Indian	4 (2.2%)	2 (4.1%)	2 (1.52%)	
Native Hawaiian/other Pacific Islander	1 (0.6%)	0 (0%)	1 (0.76%)	
More than one	5 (2.76%)	2 (4.1%)	3 (2.27%)	
Unknown	1 (0.6%	1 (2.0%)	0 (0.0%)	
Clinical presentation				
Acute appendicitis	77 (42.5%)	18 (36.7%)	59 (44.7%)	0.40
Ovarian metastases (percentage of female patients*)	40/90 (44.4%)	16/27 (59.3%)	24/63 (38.1%)	0.06
Histology				0.27
Mucinous adenocarcinoma	80 (44.2%)	24 (49.0%)	56 (42.4%)	
Goblet cell adenocarcinoma	76 (42.0%)	21 (42.9%)	55 (41.7%)	
Colonic-type (nonmucinous) adenocarcinoma	14 (7.7%)	2 (4.1%)	12 (9.1%)	
Signet ring cell	7 (3.9%)	0 (0%)	7 (5.3%)	
Poorly differentiated adenocarcinoma	4 (2.2%)	2 (4.1%)	2 (1.5%)	
Lymph node disease				0.99
Present	59 (32.6%)	16 (32.7%)	43 (32.6%)	
Not present (N0)/could not be assessed (Nx)	122 (67.4%)	33 (67.4%)	89 (67.4%)	
Lymphovascular invasion (*N *= 176; % of *N*)	77 (43.8%)	21/49 (42.9%)	56/127 (44.1%)	0.99
Perineural invasion (*N *= 176; % of *N*)	82 (46.6%)	21/49 (42.9%)	61/127 (48.0%)	0.61
Grade				0.99
Grade 1	48 (26.5%)	13 (26.5%)	35 (26.5%)	
Grade 2	55 (30.4%)	15 (30.6%)	40 (30.3%)	
Grade 3	78 (43.1%)	21 (42.9%)	57 (43.2%)	
Disease spread at diagnosis				0.50
Locoregional (M0)	76 (42.0%)	23 (46.9%)	53 (40.2%)	
Metastatic disease	105 (58.0%)	26 (53.1%)	79 (59.9%)	
Elevated baseline CEA (*N *= 146)	60 (41.1%)	19/41 (46.3%)	41/105 (39.1%)	0.46
Elevated baseline CA19-9 (*N *= 96)	22 (22.9%)	6/28 (22.2%)	16/69 (23.2%)	0.99
Elevated baseline CA125 (*N *= 94)	47 (50.0%)	18/30 (60.0%)	29/64 (45.3%)	0.27
Median PCI (IQR; *N *= 77)	20 (11–28)	25.5 (15.5–31; *n *= 18)	19 (11–28; *n *= 59)	0.21

EOAA early onset appendiceal adenocarcinoma, AOAA average-age onset appendiceal adenocarcinoma, IQR interquartile range, CEA carcinoembryonic antigen, CA19-9 carbohydrate antigen 19-9, CA125 cancer antigen 125, PCI Peritoneal Carcinomatosis Index; *Women who underwent oophorectomy for any indication prior to appendiceal adenocarcinoma diagnosis were excluded from the denominator

### Locoregional Disease Cohort

A total of 76 patients (EOAA = 23 [30.3%] + AOAA = 53 [69.7%]) had LRD disease at presentation. All patients with LRD in the EOAA group underwent curative-intent right hemicolectomy with lymph node dissection following initial diagnosis. Three (5.7%) patients in the AOAA group underwent appendectomy alone without subsequent right hemicolectomy. A total of 14 (60.9%) patients in the EOAA group and 23 (43.4%) patients in the AOAA group (*p *= 0.16), received adjuvant chemotherapy.

**Fig. 2:**
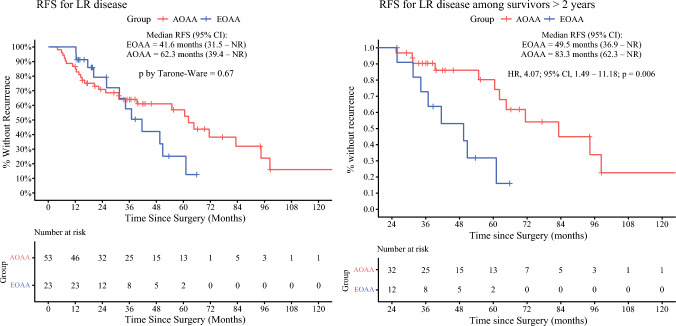
Kaplan–Meier plots for recurrence-free survival in patients with locoregional disease at presentation Tarone–Ware test was utilized due to crossing of survival curves and violation of proportional hazards assumption RFS recurrence-free survival, AOAA average-age onset appendiceal adenocarcinoma, EOAA early onset appendiceal adenocarcinoma, NR not reached, HR hazard ratio, CI confidence interval

Median follow-up among patients without recurrence/death (*n *= 36) at the time of analysis was 35.5 months (interquartile range [IQR], 19.2–43.7). There was no significant difference in RFS between the two groups (Fig. 2). Among patients who had no recurrence in the initial 2 years following surgery and had follow-up data available for more than 2 years (*N *= 44; conditional survival analysis), the risk of recurrence was higher in the EOAA group (Fig. 2). Kaplan–Meier survival plots for the two comparisons are shown in (Fig. 2). Further conditional survival analyses at the 1−(*N *= 69; median RFS, EOAA = 41.6 months versus AOAA = 71.5 months;* p* = 0.03) and 3-year (*N* = 33; median RFS, EOAA = 50.8 months versus AOAA = 83.3 months;* p* = 0.01) marks also revealed significant differences in the risk of recurrence between the two groups, with the EOAA group showing a higher risk of recurrence.

Relevant to current clinical surveillance practices (see Discussion), we further calculated the 5-year RFS percentage across the whole cohort, which was 47.7% (EOAA = 25.2%; AOAA = 56.9%). Among 15 (19.7%) patients in the LRD cohort who had no recurrence in 5 years following surgery and had follow-up data available for more than 5 years, 9 (60% of 15) patients eventually had disease recurrence (1/2 with EOAA, 8/13 with AOAA), with 8 of these recurrences occurring between 5 years and 10 years of surgery.

### Metastatic/Recurrent Disease Treatment Patterns

Overall, 139 patients (EOAA = 36 [25.9%] + AOAA = 103 [74.1%]) were included for the metastatic/recurrent disease treatment patterns and survival outcomes subanalysis.

Treatment patterns were compared (Table 2) and revealed that eight patients with EOAA received non-fluorouracil (non 5-FU) based systemic treatments (including but not limited to TAS-102, regorafenib, immunotherapy, and experimental agents) in any line of treatment versus six in the AOAA group (8/36, 22.2% versus 6/103, 5.8%;* p* = 0.009). A total of 24 patients in the EOAA group received bevacizumab in any line of treatment as compared with 49 in the AOAA group (24/36, 66.7% versus 49/103, 47.6%;* p* = 0.055), while 10 patients with EOAA received three or more lines of systemic therapy versus 14 in the AOAA group (10/36, 27.8% versus 14/103, 13.6%;* p* = 0.07). Differences in the three treatment approaches described previously were further explored using multivariable logistic regression analysis with adjustments for histology (GCA versus non-GCA), grade, lymph node disease, and survival/follow-up time. Multivariable logistic regression revealed that EOAA was associated with significantly higher odds of receiving bevacizumab (OR, 2.39; 95% CI, 1.07–5.62;* p* = 0.04) and/or non 5-FU based systemic treatments (OR, 5.05; 95% CI, 1.58–17.22;* p* = 0.007). Results from univariable and multivariable regression analyses are presented in Supplementary (Table 2).

**Table 2:** Comparison of select treatment patterns in the study population

Characteristic	Entire Cohort(*N *= 139)	EOAA(*N *= 36)	AOAA(*N *= 103)	*p*-Value
≥ 2 lines of systemic therapy given	69 (49.6%)	18 (50.0%)	51 (49.5%)	0.99
≥ 3 lines of systemic therapy given	24 (17.3%)	10 (27.8%)	14 (13.6%)	0.07
Triplet chemotherapy utilized in any line*	29 (20.9%)	5 (13.9%)	24 (23.3%)	0.34
Bevacizumab utilized	73 (52.5%)	24 (66.7%)	49 (47.6%)	0.055
Non-FU-based systemic therapy**	14 (10.1%)	8 (22.2%)	6 (5.8%)	0.009
CRS in any line	101 (72.7%)	28 (77.8%)	73 (70.8%)	0.52
Multiple CRS performed	20 (14.4%)	7 (19.4%)	13 (12.6%)	0.41
CC0/CC1 achieved in any line (among patients who underwent CRS; *N *= 101)	83 (82.2%)	21/28 (75.0%)	62/73 (84.9%)	0.26

EOAA = Early onset appendiceal adenocarcinoma, AOAA average-age onset appendiceal adenocarcinoma, FU fluorouracil, CRS cytoreductive surgery, CC completeness of cytoreduction score; *Triplet chemotherapy is mFOLFIRINOX, which includes three cytotoxic chemotherapeutic agents; **Includes any systemic therapy other than standard regimens, including but not limited to TAS-102, regorafenib, immunotherapy, and experimental agents

### Metastatic/Recurrent Disease Overall Survival Analysis

**Fig. 3:**
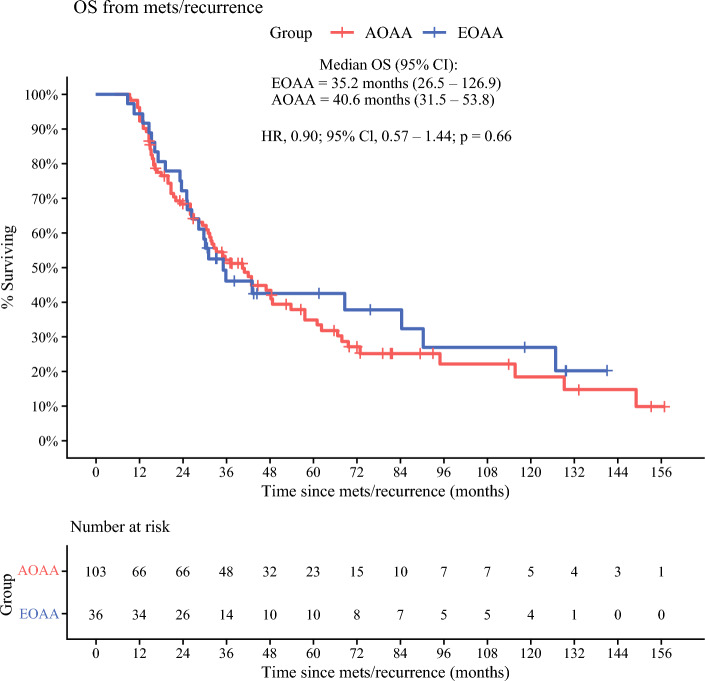
Kaplan–Meier plot for overall survival in patients with metastatic/recurrent disease, calculated from date of metastatic/recurrent disease diagnosis OS overall survival, AOAA average-age onset appendiceal adenocarcinoma, EOAA early onset appendiceal adenocarcinoma, HR hazard ratio, CI confidence interval

Overall survival from the date of metastatic/recurrent disease diagnosis was analyzed across the eligible subcohort (*N* = 139). Median follow-up among survivors was 48 months (IQR, 36.8–81.3). There was no significant difference in overall survival (OS) (Fig. 3) between the EOAA and AOAA groups. Subgroup analyses were subsequently performed on the basis of select demographic and clinicopathological characteristics, and no significant differences in OS were seen in any subgroup (Supplementary Table 3). Multivariable analysis for OS across the whole cohort revealed metachronous metastases (versus synchronous), grade 3 disease (versus grade 1), perineural invasion (versus absent), cytoreductive surgery (versus nonsurgical candidates), and use of bevacizumab (versus not utilized) to be significantly associated with OS (Supplementary Table 4).

### Next-Generation Sequencing Findings

NGS results for tumor somatic mutation testing were available in 76 patients (EOAA = 21 [27.6%] + AOAA = 55 [72.4%]). The most frequently altered genes were *KRAS* (35/76, 46.1%), *GNAS* (25/76, 32.9%), *TP53* (17/76, 22.4%), and *SMAD4* (13/76, 17.1%). *TP53* mutations were more frequent in the EOAA group with a nonsignificant difference in proportions (8/21, 38.1% versus 9/55, 16.4%;* p *= 0.06). No other notable differences in the frequencies of somatic mutations detected through NGS were observed (Supplementary Table 5). Data regarding microsatellite instability (MSI) status were available for 63 (EOAA = 17; AOAA = 46) patients, and all of these patients were found to have microsatellite-stable disease.

## Discussion

To the best of our knowledge, this is the first study of its kind exploring clinical outcomes and treatment patterns in EOAA and comparing them to an average-age onset cohort. The median age of diagnosis in our cohort for the EOAA group was 42.1 years, which is similar to the mean age at cancer diagnosis of 37.5 years (standard deviation [SD], 8.8) among patients with early onset ACs as reported by Gibbs et al., in a heterogeneous cohort.^13^ The median age at diagnosis for EOAA in our cohort was similar to early onset colorectal cancer, which is estimated to be between 44 years and 45 years.^23,24^ Current US Preventive Services Task Force guidelines recommend screening for colorectal cancer (CRC) starting at 45 years of age.^25^ Although AA is infrequently diagnosed by colonoscopy, the reported age at onset for early onset ACs raises concern, and there is a need for developing optimal surveillance practices, especially given the rapidly increasing incidence of ACs and the fact that early onset ACs represent the fastest rising early onset gastrointestinal cancer subtype.^3,26^

A considerable percentage of female patients presenting with ovarian metastases across our entire study cohort was notable. These proportions where numerically higher than CRC (3–5%), where a higher incidence of ovarian metastases has been reported in premenopausal women.^27^ This raises significant concern for ovarian masses, especially in the premenopausal patient with ACs, including consideration of appropriate fertility options, and discussions regarding prophylactic oophorectomies.^21^

Many patients with LRD developed late recurrences beyond 2 years, with nearly 60% of the whole cohort developing recurrences after 5 years following surgical resection. This phenomenon has not been described previously and creates significant challenges for surveillance and survivorship. It should be noted, however, that this finding may in part reflect the composition of our cohort, as all patients were seen at our institution, an academic referral center, which may have introduced ascertainment bias. Currently, we advocate for all patients to enroll in survivorship programs and often continue surveillance for up to 10 years.^18^ Systematic investigation of surveillance modalities, including circulating tumor DNA (ctDNA),^28^ remain critical to developing evidence-based recommendations.

In patients with metastatic/recurrent disease, patients with EOAA often received more aggressive and nonstandard therapies and yet showed no significant difference in survival. This likely indicates the aggressiveness of the disease, which may be in part due to the differences in somatic mutation profiles noted in this cohort;^10^ however, it could also be explained by younger patients possibly being in better overall health, more willing to pursue aggressive treatment, or a number of other contributing factors such as current treatment options not showing satisfactory efficacy. The potential implications of more intensive treatment, including toxicities and diminished quality of life, should carefully be considered by clinicians prior to rendering treatment decisions in younger patients.^29^ Encouraging the enrollment of patients in clinical trials is critical to advance understanding of the care of such patients.

Although favorable overall survival and progression-free survival outcomes have been previously reported in retrospective studies with the use of bevacizumab in Acs,^30^ we found bevacizumab use to be associated with worse survival outcomes in our cohort. It should be noted that the report by Choe et al. suggesting improved prognoses with bevacizumab use also included patients with “pseudomyxoma peritonei” and did not appear to include goblet cell adenocarcinomas,^30^ while our study included patients with adenocarcinomas only. Although ascertainment bias in our cohort may have influenced outcomes, given our findings that patients with EOAA were more likely to receive bevacizumab, it is paramount for clinicians to consider the potential survival and toxicity implications of bevacizumab use for these patients, especially considering the limited evidence supporting its use in patients with AA.

A signal in our data suggesting younger female patients may present with a higher frequency of ovarian metastases at diagnosis, along with our findings indicating more intensive treatment patterns, points toward EOAA being a clinically distinct entity from AOAA. Despite the overall rarity of AA, these findings, along with the rising rate of incidence of early onset ACs, underscore the unmet need for larger and prospective studies to help define optimal clinical management strategies for this potentially distinct subtype of AA.

Our results should largely be considered signals for future research. While our cohort reports a rare subset of patients within a rare disease, the limited sample size poses challenges for generalizability. Patients were selected for review through a convenience sample, which limits the external validity of our results. Our study cohort is enriched by patients who received sufficient clinical follow-up and extensive treatment at our center. As our study was intended to be exploratory in nature, no adjustment was made for multiple comparisons, and hence, our findings, particularly *p*-values approaching but not reaching conventional significance thresholds, should be interpreted with caution. Nevertheless, our study is unique in sharing real-world treatment patterns and clinical outcomes in this rare cohort of patients. Acknowledging this, several findings from our dataset warrant further investigation to help accurately define outcomes and optimize treatment strategies for this patient population.

## Conclusions

Female patients with EOAA may be more likely to present with ovarian metastases as compared with their AOAA counterparts. Patients with EOAA with locoregional disease have differential risks of disease recurrence after surgical resection, particularly among patients who remain recurrence-free for 2 years after surgical resection, with some patients experiencing recurrence even after 5 years following surgery. Patients with EOAA may receive more intensive systemic treatment than patients with AOAA, but OS does not differ significantly between the two groups. Further investigations into defining EOAA as a distinct disease entity within this rare disease are warranted.

## Supplementary Information

Below is the link to the electronic supplementary material.

Supplementary file1 (DOCX 78 KB)
