# Supplementary material for: Survival Outcomes in Early Onset Appendiceal Adenocarcinoma (EOAA)
Source: Ann Surg Oncol. 2026 Jun 6;33(9):8546–54. doi: 10.1245/s10434-026-19855-z (PMC13452864; doi:10.1245/s10434-026-19855-z)
Supplement: Supplementary file 1 [file 10434_2026_19855_MOESM1_ESM.docx]

| **Supplementary Table 1: Logistic Regression for Odds of Presence of Ovarian Metastases at Presentation (among female patients with ovaries at time of diagnosis).** | | | | |
| --- | --- | --- | --- | --- |
| **Characteristic** | **Univariable OR (95% CI)** | **p** | **Adjusted/Multivariable OR (95% CI)** | **P** |
| EOAA (vs. AOAA) | 2.36 (0.95 – 6.07) | 0.07 | 2.36 (0.93 – 6.20) | 0.07 |
| Non-GCA histology (vs. GCA) | 1.46 (0.62 – 3.48) | 0.39 | 1.51 (0.60 – 3.87) | 0.38 |
| Grade | | | | |
| Grade 1 | REF |  | REF |  |
| Grade 2 | 1.13 (0.35 – 3.72) | 0.84 | 0.99 (0.29 – 3.43) | 0.99 |
| Grade 3 | 1.07 (0.38 – 3.09) | 0.90 | 1.39 (0.41 – 4.83) | 0.60 |
| Lymph node disease present (vs. absent) | 0.67 (0.27 – 1.65) | 0.39 | 0.58 (0.20 – 1.64) | 0.31 |
| EOAA = Early Onset Appendiceal Adenocarcinoma; AOAA = Average-age Onset Appendiceal Adenocarcinoma; GCA = Goblet Cell Adenocarcinoma. | | | | |

| **Supplementary Table 2: Results from univariable and multivariable/adjusted logistic regression analysis for treatment patterns with p-values below 0.1 (for EOAA vs. AOAA) using Fischer’s Exact test.** | | | | |
| --- | --- | --- | --- | --- |
| **Characteristic** | **Univariable OR (95% CI)** | **P** | **Adjusted/Multivariable OR (95% CI)** | **P** |
| **Use of ≥ 3 lines of systemic therapy** | | | | |
| EOAA (vs. AOAA) | 2.45 (0.95 - 6.13) | 0.057 | 2.55 (0.97 – 6.65) | 0.054 |
| Non-GCA histology (vs. GCA) | 1.56 (0.62 – 4.31) | 0.36 | 2.17 (0.75 – 6.80) | 0.16 |
| Grade |  |  |  |  |
| Grade 1 | REF |  | REF |  |
| Grade 2 | 1.07 (0.28 – 4.56) | 0.92 | 2.02 (0.41 – 12.33) | 0.41 |
| Grade 3 | 1.59 (0.51 – 6.05) | 0.45 | 5.17 (1.01 – 36.60) | 0.07 |
| Lymph node disease present (vs. absent) | 0.87 (0.33 – 2.15) | 0.77 | 0.72 (0.24 – 2.08) | 0.55 |
| Survival/follow up time | 1.01 (0.99 – 1.02) | 0.34 | 1.01 (0.99 – 1.03) | 0.17 |
| **Use of bevacizumab** | | | | |
| EOAA (vs. AOAA) | 2.20 (1.00 – 5.00) | 0.051 | 2.39 (1.07 – 5.62) | 0.04 |
| Non-GCA histology (vs. GCA) | 0.72 (0.36 – 1.43) | 0.35 | 0.91 (0.41 – 2.01) | 0.81 |
| Grade |  |  |  |  |
| Grade 1 | REF |  | REF |  |
| Grade 2 | 1.49 (0.57 – 3.94) | 0.42 | 1.05 (0.36 – 3.08) | 0.93 |
| Grade 3 | 1.95 (0.82 – 4.80) | 0.14 | 1.13 (0.37 – 3.44) | 0.83 |
| Lymph node disease present (vs. absent) | 1.83 (0.91 – 3.74) | 0.09 | 1.43 (0.64 – 3.22) | 0.39 |
| Survival/follow up time | 0.989 (0.979 – 1.00) | 0.051 | 0.99 (0.98 – 1.002) | 0.14 |
| **Use of non-FU based systemic therapy** | | | | |
| EOAA (vs. AOAA) | 4.62 (1.49 – 15.11) | 0.008 | 5.05 (1.58 – 17.22) | 0.007 |
| Non-GCA histology (vs. GCA) | 1.08 (0.35 – 3.71) | 0.89 | 1.16 (0.32 – 4.48) | 0.83 |
| Grade |  |  |  |  |
| Grade 1 | REF |  | REF |  |
| Grade 2 | 1.88 (0.37 – 13.79) | 0.47 | 2.19 (0.36 – 19.83) | 0.43 |
| Grade 3 | 1.52 (0.34 – 10.68) | 0.61 | 2.41 (0.36 – 25.17) | 0.40 |
|  |  |  |  |  |
| Lymph node disease present (vs. absent) | 0.69 (0.18 – 2.18) | 0.54 | 0.49 (0.11 – 1.94) | 0.33 |
| Survival/follow up time | 0.99 (0.98 – 1.01) | 0.97 | 0.99 (0.98 – 1.02) | 0.94 |
| EOAA = Early Onset Appendiceal Adenocarcinoma; AOAA = Average-age Onset Appendiceal Adenocarcinoma; FU = Fluorouracil; *Includes any systemic therapy other than standard regimens, including but not limited to TAS-102, regorafenib, immunotherapy, and experimental agents; NB: Adjusted/Multivariable results are odds ratios adjusted for histology (goblet-cell vs. non-goblet cell), grade, lymph node disease, and survival/follow up time. | | | | |

| **Supplementary Table 4: Univariable and Multivariable overall survival analyses** | | | | |
| --- | --- | --- | --- | --- |
| **Variable** | **Univariable Cox PH analysis** | | **Multivariable Cox PH analysis (N = 135)** | |
|  | HR (95% CI) | p-value | HR (95% CI) | p-value |
| EOAA (vs. AOAA) | 0.90 (0.57 – 1.44) | 0.66 |  |  |
| Male SAAB (vs. Female) | 1.48 (0.98 – 2.24) | 0.06 |  |  |
| Non-White/Unknown Race (vs. White) | 0.89 (0.56 – 1.41) | 0.62 |  |  |
| Metachronous metastases (vs. synchronous) | 2.70 (1.72 – 4.24) | <0.001 | 2.19 (1.23 – 3.90) | 0.008 |
| GCA (vs. NGCA) | 1.62 (1.06 – 2.46) | 0.03 | 0.68 (0.37 – 1.23) | 0.20 |
| Lymph Node Disease (vs. Not Present/Not Assessed) | 2.50 (1.64 – 3.81) | <0.001 | 1.08 (0.65 – 1.79) | 0.77 |
| Grade | | | |  |
| Grade 1 | REF |  | REF |  |
| Grade 2 (vs. Grade 1) | 2.26 (1.09 – 4.70) | 0.03 | 1.04 (0.45 – 2.41) | 0.93 |
| Grade 3 (vs. Grade 1) | 4.94 (2.58 – 9.46) | <0.001 | 2.49 (1.08 – 5.71) | 0.03 |
| Lymphovascular Invasion (vs. Absent; N = 135) | 3.19 (2.05 – 4.98) | <0.001 | 1.72 (0.99 – 2.96) | 0.052 |
| Perineural Invasion (vs. Absent; N = 135) | 2.93 (1.88 – 4.58) | <0.001 | 2.17 (1.20 – 3.95) | 0.01 |
| CRS in any line (vs. no CRS) | 0.22 (0.14 – 0.33) | <0.001 | 0.33 (0.20 – 0.54) | <0.001 |
| Triplet chemotherapy in any line* | 1.35 (0.82 – 2.21) | 0.24 |  |  |
| ≥ 3 lines of systemic therapy | 1.14 (0.71 – 1.85) | 0.58 |  |  |
| Bevacizumab in any line | 1.78 (1.17 – 2.72) | 0.008 | 1.56 (1.00 – 2.44) | 0.049 |
| PH = Proportional Hazards; HR = Hazard Ratio; CI = Confidence Interval; EOAA = Early Onset Appendiceal Adenocarcinoma; AOAA = Average-age Onset Appendiceal Adenocarcinoma; SAAB = Sex Assigned at Birth; GCA = Goblet Cell Adenocarcinoma; NGCA = Non-Goblet Cell Adenocarcinoma; CRS = Cytoreductive Surgery; CC = Completeness of Cytoreduction score; *Triplet chemotherapy is mFOLFIRINOX which includes three cytotoxic chemotherapeutic agents; | | | | |

| **Supplementary Table 5: Frequencies of mutations detected through NGS stratified by age at onset for most common mutations** | | | | |
| --- | --- | --- | --- | --- |
| **Gene** | **Entire Cohort (N = 76)** | **EOAA (N = 21)** | **AOAA (N = 55)** | **p-value** |
| *KRAS* | 35 (46.1%) | 11 (52.4%) | 24 (43.6%) | 0.61 |
| *KRAS G12X* | 31 (40.8%) | 11 (52.4%) | 20 (36.4%) | 0.30 |
| *GNAS* | 25 (32.9%) | 7 (33.3%) | 18 (32.7%) | 0.99 |
| *TP53* | 17 (22.4%) | 8 (38.1%) | 9 (16.4%) | 0.06 |
| *SMAD4* | 13 (17.1%) | 4 (19.1%) | 9 (16.4%) | 0.75 |
| EOAA = Early Onset Appendiceal Adenocarcinoma; AOAA = Average-age Onset Appendiceal Adenocarcinoma; | | | | |

**Supplementary Figure 1: Selection of records for metastatic/recurrent disease care patterns and overall survival analyses.** EOAA = Early Onset Appendiceal Adenocarcinoma; AOAA = Average-age Onset Appendiceal Adenocarcinoma.
